# Supplementary material for: Are carbon emissions trading and green financial instruments synergistic? -Comprehensive quantitative research based on content analysis
Source: PLoS One. 2024 Mar 7;19(3):e0298601. doi: 10.1371/journal.pone.0298601 (PMC10919601; doi:10.1371/journal.pone.0298601)
Supplement: S1 Data — (ZIP) [file pone.0298601.s001.zip › Data/地级市Z_F工作报告.zip!/╣π╢1⁄2/╟σ╘╢╩╨/└┤╫╘╣1⁄2╓┌║┼í╛┬φ┐╦╩2╛▌═°í┐_2010.docx]

各位代表：

我代表市人民政府向大会报告政府工作，请予审议，并请政协各位委员和其他列席人员提出意见。

2009年工作回顾

2009年是我市积极应对国际金融危机、经济社会发展成效显著的一年。一年来，市政府在省委、省政府和市委的正确领导下，在市人大和市政协的监督支持下，深入贯彻落实科学发展观，全面对接《珠江三角洲地区改革发展规划纲要》（下称《规划纲要》），以“三促进一保持”为中心，以“保增长、扩内需、调结构、促和谐”为主线，迎难而上，危中寻机，市五届人大四次会议确定的各项预期目标顺利完成。

——经济增速实现全省“六连冠”。完成生产总值855.2亿元、增长16.6%，规模以上工业增加值490.7亿元、增长27%，地方一般预算收入50.04亿元、增长33.9%，增速均居全省首位。

——主要经济指标提前完成“十一五”规划目标。生产总值、地方一般预算收入、固定资产投资、社会消费品零售总额、实际利用外资、在岗职工年人均工资、农民年人均纯收入等七项指标提前一年实现“十一五”规划目标。

——产业结构调整成效明显。三次产业结构由上年的12.8：56.3：30.9调整为12.1：55.8：32.1。第三产业增加值274.6亿元，增长16.2%，对经济增长的贡献率达31.6%。全市引进和创建的名牌产品（商标）增至112个。工业经济效益明显提升，规模工业利润总额28.7亿元，增长1.6倍。农业产业化稳步推进，农林牧渔业总产值162.8亿元，增长9%。预计省定污染减排年度目标顺利完成。

——招商引资成果丰硕。新引进项目548个，同比增加85个；合同投资金额905.1亿元，增长19.8%；新项目实际投入资金123.3亿元，增长87.4%。

——外贸出口和利用外资实现正增长。全市外贸出口总额14.1亿美元、增长2.8%，实际利用外资5.5亿美元、增长6.7%，是全省实现双增长的五个市之一。

——县域经济实力大幅提升。南部和中部地区综合实力进一步增强，佛冈县、英德市、清新县经济综合发展力在全省67个县（市）中分别排第11、13和15位，包揽全省山区市县域经济综合发展力前三名。北部地区经济发展加快，连州市和连山县经济增速自2002年以来首次超过全市平均水平。

——人民群众得到更多实惠。市区居民人均可支配收入1.43万元，增长10.1%；农村居民人均纯收入5509元，增长12.7%。居民储蓄余额496.6亿元，增长13.7%。新增就业人数6.28万人，城镇登记失业率控制在3.1%以内。新型农村合作医疗参合率达98%。109.6万人的饮水不安全问题得到解决。131条贫困村、5万多贫困人口实现脱贫。

一年来，我们主要抓了以下工作：

（一）全力以赴扩大内需保增长。积极争取银行融资，市级到位融资金额56亿元。引导和鼓励社会投资，全社会固定资产投资841亿元，增长19.3%。中央、省扩内需项目和市重点项目加快推进，启动了“十个一批”城乡重点项目建设，清（远）连（州）公路高速化改造、武（汉）广（州）客运专线清远段建成通车；广（州）清（远）高速扩建清远段、广（州）乐（昌）高速清远段控制性工程正式动工；广（州）清（远）城际轨道、二（连浩特）广（州）高速、佛（山）清（远）从（化）高速公路前期工作基本就绪。武广客运专线清远站、秀丽公园等一批市政工程投入使用，清远水利枢纽、清远抽水蓄能电站、飞来湖防灾减灾工程等项目建设取得重大突破。出台政策引导、扩大住房消费，全市商品房销售面积288万平方米、销售金额98亿元，分别增长1.3倍和1.7倍。抓好家电、汽车、摩托车下乡和家电以旧换新工作，积极争取省扩大“家电下乡”目录。城乡消费市场畅旺，全社会消费品零售总额308.4亿元，增长23.1%。深入推进“双转移”工作，四大重点园区基础设施日趋完善。佛山（清远）产业转移园成为全省5个获得考核优秀等级的园区之一，佛山禅城（清新）产业转移园成为省级产业转移园及全国陶瓷产业转移示范基地。创新招商引资方式，突出园区招商和产业招商，着力培育主导产业，广东小康微车、蒙牛液态奶等一批优质项目落户园区。出台《关于服务企业促进经济平稳健康发展的意见》，大力清理涉企收费，安排近亿元资金扶持企业发展。积极搭建促销平台，组织企业参与各种经贸交流活动，帮助企业开拓市场，抢抓定单。全市民营企业达7631户，增长12.4%。

（二）积极推进产业结构调整优化。引导支持企业加快技术改造和技术创新，推动传统产业转型升级。全市工业技术改造投入70.8亿元，增长2.39倍；自主创新投入1.44亿元，增长41.2%。15个省部（院）产学研项目立项，新认定国家高新技术企业10家，新增省级企业技术中心2家，新增省级火炬计划特色产业基地、省级工程中心和省级农业创新中心各1家。国家水泥及制品质量监督检验中心落户我市。大力实施技术标准、品牌带动战略，参与制定并发布2项国家标准，“新亚”牌和“光”牌商标被评为中国驰名商标，实现了我市驰名商标零的突破。积极推动产业集聚，清城有色金属再生产业集群、佛冈龙山空调制冷配件产业集群被认定为省产业集群升级示范区。实施国民旅游休闲计划，全年接待游客1881万人次，增长26.4%；旅游总收入75.1亿元，增长33.5%。深化金融机构改革，市农信社统一法人工作取得阶段性进展，16家城信社退出市场工作基本完成；成立了4家小额贷款公司，全市担保公司发展到32家。城市商圈设施不断完善，义乌商贸城一期、国际汽车城首个4S店建成开业，大润发超市、华南装饰城等大型流通企业将入驻我市。积极推进节能减排节地工作，关停并转高耗能、高污染、低效益企业55家，预计单位生产总值能耗下降3.8%；新（扩）建的14间污水处理厂已投入试运行，新增污水处理能力22.5万吨/日；利用园地山坡地开发补充耕地13.8万亩，其中7.1万亩已通过省抽查验收，排全省第二位。

（三）大力发展农业农村经济。认真落实各项强农惠农政策，出台措施应对干旱严峻形势，确保了农民群众大灾之年不减收。大力推进农业产业化，省级、市级农业龙头企业分别增加3家和15家。农业产业化经营组织年销售收入55.5亿元，出口创汇2958万美元，带动32.6万农户户均增收3375元。积极推进农业标准化，新增农业地方标准12项，6个农业标准化示范区通过省的验收；新增绿色食品认证10个、无公害农产品产地认定企业18家、产品20个。新增备案（注册）农产品出口基地15家。加快农业基础设施建设，农田水利基本建设和基本农田整治顺利推进。提前14个月完成镇通建制村公路路面硬化任务。创造性开展“双到”扶贫开发，实施“双转移”与“双到”相结合的扶贫开发模式，大力推进产业吸引就业和智力扶贫。建立扶贫互助资金试点村57条，3150户农民加入互助资金组织，其中贫困户1944户。启动了高寒山区移民搬迁试点工作，共落实搬迁对象402户1608人。全市财政投入扶贫开发资金6000多万元。大力开展定单、定向和委托培训，共培训农村劳动力5.7万人，转移农村劳动力11.36万人次，其中就地就近就业6.53万人次。加快社会主义新农村建设。积极开展“万村绿”大行动，共完成自然村绿化160个，建设省级示范点14个。集体林权制度改革全面启动。

（四）切实加强和改善民生。全市财政民生投入达47.4亿元，增长30.9%。认真落实促进就业各项政策，积极帮扶大学生、农民工等群体就业，零就业家庭保持动态为零。超额完成养老、失业、医疗、工伤、生育保险扩面工作任务。社保基金累计结余25.9亿元。解决了早期离开国有企业职工参加社保、被征地农民参加养老保险、华侨农场和困难企业退休人员纳入医保等难题。城乡居民养老保险一体化试点工作加快推进。社会养老保险市级统筹经省验收达标。低保覆盖人口15.3万人，基本实现应保尽保。共完成287套、1.59万平方米的廉租房建设。完成2763户华侨农场归难侨危房改造，占总任务的87%。将农村饮水安全工程列为“一号民生工程”强势推进，经济开发区及6个县（区）提前一年完成全部建设任务。深入推进“城乡清洁工程”，城乡面貌进一步改善。狠抓重点区域污染整治，“大燕河流域环境污染问题”已摘牌。“市肺”工程建设加快推进。提前三年完成政府还贷二级公路收费站撤销工作。扎实推进镇（街）综治信访维稳中心建设，进京上访批次和人数分别下降63.1%和60.5%，社会大局保持稳定。扎实开展“安全生产年”活动，安全生产形势持续稳定好转。切实抓好出租车行业整顿，大力开展产品质量和食品药品安全专项整治，加大打假、打私、打传力度，市场经济秩序进一步规范。

（五）狠抓社会各项事业发展。教育发展实现新突破，全市义务教育规范化学校覆盖率达35.3%；高中阶段教育毛入学率77.7%，比上年提高13.8%。清城区、佛冈县、连山县率先实现“普高”目标。清城区、佛冈县成功创建成为省教育强区（县）。实施技能人才储备计划，技工教育培训规模进一步扩大。启动了省市共建现代职业教育体系改革试验区工作。代课教师“代转公”问题得到妥善解决，教师绩效工资和工资福利待遇“两相当”得到较好落实。积极推进医疗卫生体制改革，疾病预防控制、妇幼保健服务、农村（社区）卫生服务上新水平，应对甲型H1N1流感疫情科学有效。基层医疗卫生机构建设得到加强，市人民医院新院区一期工程已竣工。启动了创建国家卫生城市工作。文艺创作上新台阶，文化遗产保护工作继续加强。文化体育配套设施不断完善。成功举办第四届市运会、第八届广场文化艺术节等大型文体活动。计划生育工作受到省的表彰。民族、宗教关系保持和谐。国防建设和民兵预备役工作进一步加强。妇女、儿童、老龄、人防、气象、统计、审计、档案、方志、外事、侨务、对台、红十字、广播电视等各项工作均取得新成绩。

（六）加强民主法制和政府自身建设。积极开展深入学习实践科学发展观活动，形成应对国际金融危机、推动科学发展的强大动力。大力推进依法治市，积极开展“五五”普法和法律“六进”（进机关、进乡村、进社区、进学校、进企业、进单位）活动，自觉接受市人大及其常委会、市政协的监督，开展人大代表约见市长活动，共办理人大代表议案71件、政协提案182件。密切政府与各民主党派、工商联、人民团体、无党派人士的联系，科学民主依法决策机制进一步完善。推进机构编制实名制管理，市县两级机构改革扎实推进。政务公开取得新进展，市政府门户网站功能进一步完善，在全国同类网站评比中排名第23位。加强机关作风建设，对全市411家重点单位进行明查暗访，共受理行政效能投诉262件，办结227件。积极推进廉政建设，惩治和预防腐败体系不断完善。加强应急管理体系建设，应对突发公共事件能力有效提升。

各位代表！过去一年，我们能取得好于预期的成绩极其不易。这是中央和省正确领导的结果，是市委、市政府早决策、早行动，带领全市人民开拓进取、扎实工作的结果，是各位人大代表、政协委员监督支持的结果，是港澳台同胞和社会各界关心支持的结果，是兄弟地区尤其是对口帮扶地区热心帮助的结果。在此，我代表市人民政府，向全市各族人民，向各民主党派、人民团体、社会各界人士，向中央、省驻清单位，驻清解放军、公安干警、武警和消防官兵，向港澳台同胞、海外侨胞，以及所有参与和支持清远建设的海内外朋友，表示衷心的感谢并致以崇高的敬意！

在肯定成绩的同时，我们也清醒认识到，我市发展方式粗放、产业层次整体偏低等深层次矛盾尚未得到根本改变，经济社会发展还存在一些突出困难和问题：经济总量还比较小，加快发展任重道远；自主创新能力弱，产业竞争力不强；区域发展仍不协调，扶贫开发压力较大；资源环境约束加大，节能减排任务艰巨；落实公共服务均等化财政保障压力较大，政府服务水平有待进一步提高。我们必须正确面对困难和问题，采取有力措施切实加以解决。

2010年工作安排

2010年是我市巩固应对国际金融危机成果、夯实经济回升基础的关键之年，也是加快发展方式转变、推动经济社会又好又快发展的关键之年。今年发展的形势好于去年。特别是随着国家和省扩内需保增长政策成效的进一步显现，省委、省政府《关于促进粤北山区跨越发展的指导意见》的出台，以及我市区位优势的进一步凸显，我市经济持续快速发展的外部环境更加有利。但是，我们也要看到，发展仍面临诸多不确定因素，经济回升的基础还不牢固。因此，我们既要增强必胜信心，全力推动发展，更要增强忧患意识，科学谋划各项工作，力争经济社会发展实现新突破。

2010年工作的总体要求：深入贯彻落实科学发展观，认真落实党的十七大、十七届四中全会、中央经济工作会议、省委十届六次全会、粤北山区工作会议和市委五届九次全会精神，积极对接《规划纲要》，以转变经济发展方式为核心，以“抓提速、调结构、促转变、惠民生”为主线，大力推进“三促进一保持”，突出抓好扩大内需，增强经济发展动力；突出抓好结构调整，提升经济发展质量；突出抓好交通基础设施建设，加快融入广佛都市圈；突出抓好“双转移”工作，加快城镇化进程；突出抓好民生改善，确保社会和谐稳定；突出抓好政府自身建设，优化区域发展环境，努力提升科学发展的实力和水平，继续争当山区科学发展的排头兵。

经济社会发展的主要预期目标是：生产总值增长18%，人均生产总值增长16%；单位生产总值能耗下降、二氧化硫和化学需氧量削减量完成“十一五”规划目标；全社会固定资产投资增长18%；实际利用外资增长10%，外贸出口总额增长6%；财政一般预算收入增长20%；社会消费品零售总额增长20%；居民消费价格总水平控制在3.5%以内；城镇登记失业率控制在3.5%以内；人口自然增长率控制在8‰以内；在岗职工平均工资增长10%，农村居民人均纯收入增长10%。

同时，要积极推动服务业发展，引导和加大全社会科技投入，不断提升服务业增加值占生产总值比重、研究与发展经费支出占生产总值比重。

为实现上述目标，必须做好以下八项工作：

一、进一步扩大内需，推动经济持续快速增长

加快重点项目建设。着力解决项目推进中遇到的征地拆迁等突出问题，推动广清城际轨道、二广高速清远段、佛清从高速北段等省重点项目早日动工建设。加快启动金谷科学城、阳山粤能“上大压小”电源项目、侨远电厂扩建等一批重大项目建设。积极争取汕（头）湛（江）高速清远段、汕（头）昆（明）高速清远段、北江千吨级航道改造等项目列入省重点工程。加快推进广清高速扩建清远段、广乐高速清远段、清远水利枢纽、飞来湖防灾减灾工程、清远抽水蓄能电站、500千伏库湾输变电工程等在建项目建设。着力推进农村饮水安全工程、乡村公路等直接惠及民生的中低层次扩内需项目建设。千方百计激活民间投资，积极引导社会资金参与项目建设。

积极拓展城乡消费。组织好家电、汽车、摩托车下乡，深入推进“万村千乡市场工程”，进一步拓展农村消费市场。积极推进商贸流通市场建设，加快中汽南方国际汽车城建设，力争市农副产品批发市场主体工程完工、清远义乌商贸城全面建成。积极引导和促进房地产市场健康发展，增加普通商品住房供给，加快保障性住房建设。加强价格监管，确保价格总水平基本稳定。

加大企业帮扶力度。认真落实帮扶企业各项政策措施，适时出台新的扶持政策。建立重点企业直通车制度，支持推动优质企业上市，鼓励企业做大做强。引导金融机构创新服务，缓解中小企业融资难问题。政府采购优先向本地自主创新产品倾斜。继续落实专项资金，重点扶持中小企业技术创新、结构调整、节能减排。积极组织企业参与“广货北上”、“广货西行”等活动，继续办好清远名优产品展销推介会等各类会展、促销活动，帮助企业开拓市场。

二、加大结构调整力度，促进发展方式转变

切实提高自主创新能力。引导支持企业加快科技创新体系建设，加大技改投入力度，深化产学研结合，推动建材、有色金属、电子、生物制药等支柱产业优化升级。着力引进与支柱产业相配套的规模企业，进一步完善和延长特色主导产业链条。加快粤北水泥生产基地、国家水泥及制品质量监督检验中心建设。继续实施名牌带动战略、技术标准战略和以质取胜战略，引导和鼓励企业争创品牌，提高竞争力。加强与大专院校、科研院所的联系，加大创新型科研团队和科技带头人引进力度。切实抓好技术引进工作，高度重视技术的消化、吸收与再创新，实现“引资”与“引智”相结合。

加快发展第三产业。积极推进旅游资源整合和深度开发，大力推行旅游标准化，加快推进景区景点和旅游配套设施建设，提高旅游业发展水平。加强与珠三角、港澳地区在旅游项目投资、客源市场拓展、无障碍旅游设立、旅游信息交流等方面的合作，重点推进国际（中国·佛冈）健康养生旅游示范基地建设，做大生态休闲、民族风情特色旅游品牌。积极实施国民旅游休闲计划，加快开发高端旅游产品，延伸旅游产业链条。切实做好旅游宣传促销文章。积极引进和培育大型流通企业，加快物流业发展。大力发展面向珠三角的高端会展业，加快发展电子商务、金融、保险、服务外包等生产性服务业，积极推进会计、法律、咨询、评估等中介服务业发展，提高服务业在产业结构中的比重。

加强资源和环境保护。加快淘汰落后和过剩产能，加强重点耗能企业节能降耗专项监察，确保完成“十一五”落后产能淘汰和节能减排目标任务。积极推广使用新能源、新技术。完善污水处理厂配套管网和垃圾无害化处理设施。加快市级重点污染源在线监控系统建设。完成土地利用总体规划修编，严格土地审批制度，严守耕地保护“红线”。切实抓好补充耕地工作，加大闲置土地清理力度，严厉查处违法违规用地行为。严格落实水资源管理制度，确保饮用水源安全。加强环境执法监督，狠抓省挂牌督办的涉铅企业污染整治，加快推进新北江制药厂搬迁工作。严格项目资源环境准入，切实抓好矿产资源管理。加快“市肺”工程建设。

三、着力抓好“双转移”工作，加快城镇化进程

提升园区发展水平。进一步提高园区规划建设、招商引资和产业发展水平。围绕主动融入珠三角现代产业分工体系，着力引进世界500强企业、行业龙头企业、大型央企和民企，加快培育机械装备、汽车零配件、电子信息等主导产业，推动佛山（清远）产业转移园成为高端产业成长的重要平台，发挥示范带动作用。进一步完善佛山禅城（清新）产业转移园、民族工业园、华侨工业园配套设施，突出主导产业招商和园区招商，打造各具特色的主导产业。

积极推进产业集聚。全面开展主体功能区规划试点工作，做好“三圈四带”空间布局和产业布局，引导各主体功能区加快发展优势产业和特色产业，加快形成产业集群。积极推进清城有色金属再生产业集群、佛冈龙山空调制冷配件产业集群建设，继续抓好产业集群升级示范区、省市合作共建先进制造业基地的申报与建设工作。推动新引进工业项目进园，加快落实再生金属拆解户入园经营。

大力推动人口集聚。围绕对接广佛都市圈，科学修编城市规划，实现城乡规划、土地利用规划、产业发展规划的有效衔接，增强城镇吸纳人口集聚功能。高度重视城市中心区域规划建设，完善市区城市功能。加快户籍制度改革，促进农村人口到城镇落户。依托“双转移”，切实抓好农村劳动力技能培训和转移就业，促进农村劳动力向城镇集中。

加大城镇建设力度。积极推进县城和中心镇交通、供水、供电等基础设施建设，进一步完善配套服务功能，促进城镇建设扩容提质。结合开展旧城镇、旧厂房、旧村庄“三旧”改造，启动宜居城乡建设，加快建成一批有清远特色的宜居城镇、宜居村庄。深入推进“城乡清洁工程”，积极对接珠三角“绿道”建设，优化城乡环境。

四、深入推进“双到”扶贫开发，夯实“三农”发展基础

狠抓“双到”扶贫工作。完善“一户一策一干部”挂钩帮扶机制，大力推进产业扶贫、转移就业扶贫和智力扶贫，提高贫困户自我发展能力。发挥农业龙头企业和专业合作组织作用，进一步扩大贫困村互助资金覆盖面。广泛动员社会力量参与扶贫开发，积极推进省直机关、佛山市挂点帮扶工作，今年完成贫困人口脱贫10万人以上、100个村集体经济超过3万元以上。切实抓好清新、连南、阳山县高寒山区移民搬迁安置试点工作。加快与省扶贫网的对接，实现贫困户帮扶情况网络动态管理。

提升现代农业发展水平。继续推进“一乡一品”活动，进一步扩大名优家禽、蔬菜、水果等优势农产品基地规模，做强特色农业。加大农业科技研发、引进、推广和转化力度，积极推进农业机械化。加快农业龙头企业引进、培育，大力发展“订单农业”。加强农产品质量安全监管，推进农业标准化生产。积极发展无公害农产品和绿色食品。鼓励名优农产品申请注册原产地证明商标或集体商标。加快发展农民专业合作组织，积极培育专业化、市场化的农业社会服务体系。

加强农业基础设施建设。继续抓好省人大农田水利基本建设议案、基本农田整治、基本农田保护示范区建设等项目实施，全面完成城乡水利防灾减灾工程、病险水库除险加固任务，加快高标准农田建设，提高农业综合生产能力。积极推进农村电网改造，加快农村道路硬底化，全面完成农村饮水安全工程建设任务。

加快新农村建设。大力推进“八十村示范，千村整治”工程，进一步改变农村面貌。积极开展“万村绿”大行动。加强农村公共服务能力建设，积极推进文化、科技、卫生“三下乡”。加快农家书屋、农民健身设施等文化体育工程建设，充分发挥“岭南流动书香车”作用，提高农民生活质量。

五、深化重点领域改革，提升对外开放水平

积极推进行政管理体制改革。围绕建设为民、高效、廉洁的服务型政府，深化行政审批制度改革，实现精简审批、提速审批和“阳光审批”，切实提高行政审批效能。整合市行政服务中心资源，方便群众和企业办事。按照大部制改革实施方案，全面完成市、县两级政府机构改革任务。探索建立多元化的基本公共服务供给制度，积极推进“政府购买服务”，鼓励行业协会、人民团体、社会组织和企业参与提供公共服务。积极推进事业单位分类改革和人事制度改革，认真实施事业单位绩效工资制度。

深化财税、金融和物价改革。积极培植税源，切实加强税收征管。强化财政支出管理，着力打造制度完善、管理精细的“阳光”财政。优化财政支出结构，确保更多财力用于解决民生问题。进一步深化农村信用社改革，加快市联社组建及向商业银行过渡步伐。加强金融和担保体系建设，筹建政府政策性担保公司。推动保险业发展，培育和发展资本市场。扎实推进价格改革，加快制定垃圾处理收费、居民阶梯式电价和水价改革方案，积极推进区域公路年票互通和区域通信价格一体化工作。

稳步推进农村综合改革。认真贯彻落实市委、市政府《关于加快推进农村改革发展的意见》，稳定和完善农村基本经营制度，引导家庭经营向生产集约化方向发展。规范农村土地管理和流转机制，推动农业用地向规模经营集中。创新农村金融体制，促进城乡经济社会发展一体化。健全农村民主管理制度。积极推进县乡管理体制创新。积极稳妥推进集体林权制度改革。继续抓好华侨农场改革工作。

提高外经贸发展水平。加大重点出口企业帮扶力度，着力培育和发展自主出口品牌，扩大一般贸易比重和机电、高新技术产品出口比重，保持大宗商品和传统优势产品市场占有率。支持加工贸易企业扩大内销。继续实施“走出去”战略，借助广交会等境内外知名展会平台，充分发挥行业协会、商会的作用，积极扩大对外经贸交流合作，巩固欧美日等传统市场，开拓非洲、东盟、中东、南美等新兴市场。继续抓好出口基地建设，重点培育农产品代理出口企业，挖掘新的出口增长点。

六、高度重视改善民生，确保社会和谐稳定

完善社会保障和救助体系。抓好社会保险扩面工作，全面推进新型农村养老保险、被征地农民养老保险和城镇居民医疗保险工作。积极推进医疗保险门诊统筹。认真落实地税部门全责征收社会保险费工作。加强社会保险基金监管，完善社保基金市级统筹。建立健全社会救助、优抚安置、社会福利服务保障，实现动态管理下的应保尽保。进一步提高城乡低保标准，加大低收入群众帮扶救助力度。加快发展残疾人事业。切实加强革命老区帮扶工作。

进一步稳定和扩大就业。落实更加积极的就业政策，完善城乡统筹就业机制，积极开发公益性就业岗位，引导促进企业创造更多就业岗位。加强农民工就业服务，引导支持高校毕业生到基层、中小企业就业和自主创业。鼓励和支持劳动者就地就近就业。加强就业援助，重点帮扶“4050”人员、零就业家庭等困难群体就业。加强劳动监察和仲裁，发展和谐劳动关系。

加强和改善社会管理。完善突发公共事件应急处置机制，提高突发事件应急管理水平。加强安全生产事故隐患排查和重点行业、领域安全生产专项整治。加强法律援助，保障人民群众合法权益。妥善解决水库移民后续发展问题。加大社会矛盾纠纷排查和调解力度。进一步加强社会治安综合治理。大力整顿和规范市场经济秩序，严厉打击生产销售假冒伪劣产品行为。加强食品药品监管，抓好农产品质量安全和重大动物疫病防控工作。抓好粮食安全保障工作。

七、大力发展社会事业，提高公共服务水平

优先发展教育事业。积极推进教育事业均衡协调发展，加快义务教育规范化学校建设，加快普及高中阶段教育，确保完成“普高”任务。积极推进现代职业教育体系改革试验区建设，做强做优职业技术教育。深入实施技工教育倍增计划，加快市职业技术学院、市高级技校扩建工程、市公共培训中心、市新职校和市特殊教育学校建设。继续推进教育创强工作，提升教育质量和水平。进一步完善和落实义务教育学校绩效工资、中小学教师工资福利待遇“两相当”工作。

加快卫生事业发展。积极推进医药卫生体制改革。整合市区医疗资源，加快完善城市社区和农村三级医疗卫生服务网络，积极推进基层医疗卫生机构标准化建设。引导民营医疗机构规范发展。积极发展中医药事业。进一步抓好农村合作医疗工作。加快120急救指挥网络建设。加强重大传染病防控和免疫规划工作。扎实推进国家卫生城市创建工作。

积极推进文化、科技、体育等事业发展。抓好城乡公共文化惠民共享工程，继续完善公共文化服务网络。加快文化产业载体招商引资，精心策划组织丰富多彩的文化活动。继续抓好文化体制改革。深入开展群众性精神文明创建活动，推进国家文明城市创建工作。大力发展科技事业，积极推进“数字清远”建设。深入开展全民健身活动，做好省第十三届运动会参赛工作。加强人口计划生育服务管理。大力实施妇女儿童发展规划，确保按期完成任务。认真贯彻落实《广东省基本公共服务均等化规划纲要》，加快出台具体实施方案。继续做好国防、双拥、民族、宗教、侨务、外事、方志等工作。

科学编制“十二五”规划。坚持对接《规划纲要》，以推动经济结构优化升级、加快发展方式转变为重点，突出科学发展、协调发展和民生优先，高水平、高质量完成“十二五”规划编制工作。

八、加强政府自身建设，提升推动科学发展能力

突出抓好民主法制建设。认真落实人大及其常委会各项决议，坚持向人大及其常委会报告工作，自觉接受监督。认真接受人民政协的民主监督，主动听取各民主党派、无党派人士和人民团体的意见建议。认真办理人大议案、政协提案。加大政务公开力度，及时向市民公开政府决策、政务信息，拓宽社情民意反映渠道。深入推进市、县依法行政工作。加强行政执法监督，规范行政执法行为。切实做好行政复议工作。建立和完善信访案件倒查机制，进一步落实执法责任制和错案责任追究制。

深入推进廉政建设。进一步完善行政权力运行机制，实施廉政风险防范，全面落实党风廉政建设责任制。强化审计监督，规范土地使用权出让、建设工程招投标、产权交易、政府采购等要素市场，加强政府投资项目和专项资金管理。充分发挥电子监察系统在防治腐败中的作用。依法依纪打击和惩治腐败行为。

强化公务员队伍建设。加强调查研究，切实转变作风，提高服务水平。坚持厉行节约、勤俭办事，严格控制一般性行政经费支出。完善督查、问责机制，狠抓重点项目、重点工作落实。加强部门和行业不正之风整治，认真解决群众反映强烈的突出问题。深入开展行政效能监察，加强对机关单位，特别是窗口服务部门的明查暗访。抓好公务员理论、政策、业务、法律知识的学习，建设政治坚定、业务精通、清正廉洁、作风优良的公务员队伍。

各位代表！新的一年，转变经济发展方式、提升科学发展实力，我们面临的任务艰巨而繁重。让我们更加紧密团结在以胡锦涛同志为总书记的党中央周围，在市委的正确领导下，深入贯彻落实科学发展观，继续解放思想，开拓进取，求真务实，努力推动清远经济社会又好又快发展，加快建设大广州卫星城、环珠三角高端产业成长新区、华南休闲宜居名城！
